# Lay perceptions and illness experiences of people with type 2 diabetes in Indonesia: a qualitative study

**DOI:** 10.1080/21642850.2019.1699101

**Published:** 2019-12-10

**Authors:** Anna Wahyuni Widayanti, Susan Heydon, Pauline Norris, James A. Green

**Affiliations:** a School of Pharmacy, University of Otago, Dunedin, New Zealand; b Faculty of Pharmacy, Universitas Gadjah Mada, Yogyakarta, Indonesia; c School of Allied Health and Physical Activity for Health, Health Research Institute (HRI), University of Limerick, Limerick, Ireland

**Keywords:** Illness perceptions, coping strategy, diabetes, health behaviour, Indonesia

## Abstract

**Background:** Understanding perceptions and experiences of people with diabetes is important before establishing effective interventions. Previous research indicates that socio-cultural characteristics influence people’s views about diabetes.

**Objective:** This study aimed at understanding diabetes from the perspective of people with diabetes in the Indonesian cultural context.

**Methods:** Six focus group discussions involving 45 people with diabetes were conducted in East Nusa Tenggara and West Sumatera. The discussions were recorded, transcribed verbatim in their original language, translated into English, and analysed for common themes.

**Results:** This study showed that participants tried to understand diabetes based on their personal experiences. They also saw the disease in a broader context of cultural identity and changes in their cultural environment. In coping with the disease, three strategies were identified: seeing it as beyond their control, normalising their condition, and resignation to God. People who used the first and second methods of coping tended to have a more negative response to diabetes treatment. People with strong religious beliefs coped more positively with diabetes.

**Conclusions:** People with diabetes conceptualised the disease into their own narratives. These lay concepts influenced their strategies of coping and their behaviours in managing the disease. Understanding people’s lay perceptions and experiences are important to develop personalised strategies of diabetes management that may influence people’s responses to their disease and treatment.

## Introduction

Indonesia is the fourth most populous country and has the sixth-largest number of people with diabetes in the world (>10 million people with diabetes) (International Diabetes Federation, 2017). The increase in the number of people with diabetes in Indonesia is a result of increasing modifiable risk factors including hypertension, abdominal obesity, obesity, pre-diabetes, and smoking (Hussain, Mamun, Reid, & Huxley, 2016; Ng et al., 2006; Roemling & Qaim, 2012; Usfar, Lebenthal, Atmarita, Soekirman, & Hadi, 2010). Diabetes affects Asian people at a lower degree of obesity, and at a younger age than in other parts of the world (Yoon et al., 2006).

The vast majority of people with diabetes in Indonesia have type 2 diabetes with the average age of diagnosis at 49.7 ± 6.8 years old (Soewondo et al., 2010). Therefore, we focused on type 2 diabetes, and hereafter the term diabetes in this article refers to type 2 diabetes. Type 2 diabetes is characterised by slow onset of symptoms. Most people in Indonesia consider themselves healthy if they can perform their everyday activities without disruption. Thus, people generally seek help when symptoms hinder these (Fles et al., 2017; Pitaloka & Hsieh, 2015; Pujilestari, Ng, Hakimi, & Eriksson, 2014). Therefore, many people do not realise they have diabetes until they get complications. This has led to a high prevalence of undiagnosed diabetes in Indonesia (Pramono et al., 2010).

Moreover, most people with diabetes in Indonesia have poor glycaemic control. Consequently, complications are prevalent (Pemayun & Naibaho, 2017; Yusuf et al., 2016), and costs for managing the disease increased (Andayani, Ibrahim, & Asdie, 2010). This heavy financial burden affects not only individuals, but also the country, particularly in the newly implemented Universal Health Coverage programme (Agustina et al., 2019). The Indonesian Universal Health Coverage programme, known as the National Social Health Insurance Scheme (*Jaminan Kesehatan Nasional, JKN*) was introduced in January 2014. The insurance scheme covers most health interventions, including for diabetes, for people registered under the scheme (Agustina et al., 2019).

Health care in Indonesia, including for diabetes, is delivered by a mixed system in which government financed and privately financed health centres coexist. The government-health centres consist of community health centres (*Puskesmas*) in sub-district level and state hospitals at district and province level (Indonesia Ministry of Health, 2016). Diabetes speciality clinics are commonly located in hospitals, as they have more sophisticated laboratory facilities, specialist doctors, and a more extensive range of medications. The government has also introduced some programmes specific to non-communicable diseases including diabetes: integrated health post for managing non-communicable diseases (*Posbindu PTM*), and programme for managing chronic diseases (*Prolanis*). The *Posbindu PTM* programme focus mainly on early detection of risk factors and improving general health, while the *Prolanis* programme focuses on improving outcomes for people with diabetes and hypertension.

Although efforts have been made to early detect and improve outcomes of people with diabetes, an effective diabetes treatment requires people with diabetes to make a variety of behavioural changes including dietary adjustment, exercise, and medication. Thus, they themselves are the key component in diabetes management. Studies conducted in the high-income countries have demonstrated that people’s perceptions about diabetes influenced their self-management behaviours and outcomes (Alzubaidi, Mc Narmara, Kilmartin, Kilmartin, & Marriott, 2015; Broadbent, Donkin, & Stroh, 2011). For example, Alzubaidi et al. (2015) found that people with diabetes with Arabic cultural backgrounds living in Australia had less accurate illness and treatment beliefs about diabetes compared to Caucasian participants, and this was significantly correlated with poorer adherence to treatment. Therefore, understanding the perceptions and experiences of people with diabetes is important before establishing interventions. Studies involving people with diabetes with various cultural backgrounds have also shown that socio-cultural characteristics influenced their views about diabetes (Amarasekara, Fongkaew, Turale, Wimalasekara, & Chanprasit, 2014; Mendenhall et al., 2016; Suparee, McGee, Khan, & Pinyopasakul, 2015). For example, some people with diabetes in Sri Lanka and Thailand viewed that their diabetes was due to bad *karma* (bad deeds) (Amarasekara et al., 2014; Suparee et al., 2015), and some people with diabetes in India viewed that having diabetes was their fate (Mendenhall et al., 2016). This underlines the need to recognise people’s perceptions about diabetes in different cultural contexts.

Research on this area in the Indonesian context, however, is scarce. This study aimed to understand perspectives and experiences of people with diabetes in the Indonesian context.

## Materials and methods

### Study design

A qualitative study design with Focus Group Discussions (FGDs) was used. Within the group discussion, people might feel less threatened and might also be prompted to talk about other issues than when they are individually interviewed (Barbour, 2010).

### Study locations

The study was conducted in East Nusa Tenggara and West Sumatera Provinces. Both provinces are located outside Java Island, the most developed region of the country. The demographics of each province are markedly different. The majority of people in West Sumatera are originally from a single, large ethnic group, *Minangkabau*, who resemble Malay people. Islam is the predominant religion in West Sumatera (Statistics Indonesia, 2011). In contrast, the people in East Nusa Tenggara have heritage from many different small ethnic groups, and most of their origin ethnically and culturally lay in a transition border from Asia to Australia and Micronesia. The predominant religion in this province is Catholic (Statistics Indonesia, 2011). Therefore, gathering data in those provinces is likely to contribute rich information and insight about people’s perspectives from different socio-cultural backgrounds.

Some districts were further selected as study locations. Kupang city, Manggarai and Manggarai Timur were districts selected in East Nusa Tenggara; and Pariaman city and Solok were districts selected in West Sumatera.

### Participants

Potential participants were screened for eligibility by health professionals at the community health centres and the researcher (first author), from the list of diabetes patients registered at the community health centres. The inclusion criteria were: diagnosed with diabetes by health professionals (physicians), and able to comprehend information and respond to the questions in Bahasa Indonesia or the local ethnic language.

Eligible participants were invited to participate verbally. Either health professionals or the researcher invited eligible participants when they visited community health centres, at their home or by phone call. In study locations with limited patient data, participants were sought by snowball sampling (asking people with diabetes or community health centre staff about their diabetic friends or relatives) and announcing the study after a Sunday service at a church in East Nusa Tenggara. All eligible participants who could be reached agreed to participate.

### Data collection

Data were collected from September to October 2015. The FGD question guide was developed in English and translated into *Bahasa Indonesia*, the Indonesian national language (Table 1). This article presents the findings related to participants’ perceptions and experiences, and aiming at answering questions number 1–3.

**Table 1.** Focus group discussion guide in English.

Number	Questions
1	How were you first diagnosed with diabetes?
2	What do you think about diabetes?
3	What do you think is the cause of diabetes?
4	Could you please tell us your experiences in managing your diabetes after being diagnosed?
5	In terms of medicines, what type of medicines are you taking?
6	Is there any medicine that you are taking other than medicines from your doctors?

Six FGDs were conducted in *Puskesmas* or in a participant’s house, and they lasted for about two hours. As an appreciation to the participants, random blood glucose, cholesterol, and uric acid measurements were provided to all participants at the location of the discussion, before the discussion was started. In the study locations, most people with diabetes only have their blood measured when they visit health facilities. Most of them do not have their own device for measuring blood glucose levels. Therefore, people were pleased when they were given blood measurement services, as shown with their expression: ‘So, thank God, that I had my sugar level checked today’.

All discussions were conducted in *Bahasa Indonesia*. Although most Indonesians speak the language of their local ethnic group for everyday conversation, *Bahasa Indonesia* is widely used for communicating with people from outside their own ethnic groups. The main investigator in this study (first author) moderated the discussion. She is an Indonesian, but ethnically Javanese, which is different from the participants involved. In some cases, the participants answered in a combination of *Bahasa Indonesia* and a local language. Research assistants who were native speakers of the participants’ local language helped as note-takers and translators.

The discussions were recorded and transcribed verbatim in *Bahasa Indonesia*. These transcripts were then translated into English by the main investigator (the first author) who was assisted by another person who is fluent in both languages. We translated the transcripts separately. Then we exchanged the translated transcripts and compared them with the transcripts in *Bahasa Indonesia*. We indicated sentences, phrases, and words that might have a different meaning in each language and discussed them to find the closest translation. Some words that were specific in Indonesian or the local languages were left without translation.

### Data analysis

Inductive qualitative content analysis (Elo & Kyngäs, 2008) was applied to allow us to be as open as possible to unexpected findings as very few similar studies had previously been conducted in the Indonesian context. Nvivo software (Nvivo Pro version 11) was used in data analysis. To increase the trustworthiness of the findings, the categories and the content of categories were checked by the second, third, and fourth authors. Discussions involving all authors were also had to finalise the coding process. In reporting this qualitative study, we complied with the Standard for Reporting Qualitative Research (O’Brien, Harris, Beckman, Reed, & Cook, 2014).

### Ethics approval

Ethics approval was sought through and granted by the University of Otago Human Ethics Committee, New Zealand (H15/057). In study locations, the research approval letters were issued by *Kantor Pelayanan Terpadu Satu Pintu* (The Integrated Office of Community Service). The information sheet for the study was read and explained to the participants with written informed consent recorded.

## Results

### Demographic characteristics

Forty-five people with diabetes participated in six FGDs (Table 2). Each FGD was attended by 6–10 participants. Some participants who were older or had problems in reaching the discussion sites (for example, could not ride a motorbike) came to the FGDs with a family member (her/his daughter or his wife). The family members sometimes helped the participants answer the questions and were involved in the discussions. The majority of participants were aged between 40 and 59 years old, female, and housewives.

**Table 2.** Demographic characteristics of participants.

Demographic characteristics	Categories	Number of participants
Age (year)	20–39	2
	40–59	34
	60–79	9
Gender	Female	31
	Male	14
Education	Elementary school	5
	Junior high school	7
	Senior high school	15
	Tertiary education	18
Occupation	Housewives	23
	Employees	3
	Civil servants	14
	Retired	5

### Lay perception of diabetes

#### Local terms and lay knowledge of diabetes

Lay people referred to diabetes as ‘*penyakit gula’* (sugar disease), or ‘*penyakit kencing manis’* (sweet pee disease). From the perspective of participants, the term ‘sugar disease’ describes the cause of diabetes, while the term ‘sweet pee disease’ describes diabetes symptom. Some participants observed having sweet pee as indicated by ants gathered around it.

When I saw my urine attracted ants, and it was brown, I checked. It [blood sugar level] was 318 [mg/dL] (17.7 mmol/L) at that time  … (FGD Pariaman).

Other than local terms, participants classified diabetes into: ‘*penyakit gula basah’* (wet sugar disease); and ‘*penyakit gula kering’* (dry sugar disease). People differentiated these two types based on characteristics of the people with diabetes. People were perceived to have ‘wet sugar disease’ if they were getting fatter after contracting the disease and/or experienced unhealed wounds, and were perceived to have ‘dry sugar disease’ if they were getting thinner after having the disease and did not commonly experience wounds.

I have a family member in Jakarta, he experienced extreme weight loss. He is getting thinner and thinner. Is that true that he suffered from dry sugar disease? (FGD Kupang city).

Yes, somebody in my neighbour also has sugar disease, but she could not come here, her feet got wounded … Yes, that’s wet sugar disease (FGD Solok).

As far as we know, this distinction between wet and dry forms of diabetes is not used by people in Western countries.

#### Beliefs about diabetes causality

Participants attributed diabetes to hereditary factors, eating habits, and lifestyle changes. However, they elaborated these causal beliefs further with their own personal explanation, based on their experiences. In some cases, where they could not find personally relevant explanations, they started questioning why they got the disease, and in the end, they made their own tentative conclusions about that. As can be seen in quotes below, they often use word ‘*mungkin’* in the Indonesian language, which translates as ‘perhaps’ or ‘probably’, to show uncertainty.

But sometimes I think why I got the disease, what’s the symptoms of diabetes, that’s my questions … I work in training department, and my job is to prepare food for the training programmes, so perhaps [because] I always taste the food (FGD Kupang city).

Participants who believed that heredity was the cause of their diabetes also had family members with diabetes. In the Indonesian context, the term ‘family’ or ‘*keluarga*’ includes extended families and other relatives. These participants also developed a sense of vulnerability to the disease for their future generations. Some participants who perceived that their efforts to manage the disease had failed eventually believed heredity was a cause.

I have a question though, if our parents have [sugar disease], do their children definitely will also have it? All of their children? (FGD Manggarai).

But, we got these diseases, sugar disease and high blood pressure, from our ancestors, it is inherited, all of my family, my grandmother, my aunt, all of them died of this disease. So, I have had the disease for more than 4 years, but in the last 3 years I took medicines. Then, just recently, about 2 months, I have given injection,  …  insulin. But everything just the same, my sugar never goes down. Ya, perhaps that because my eating habit, but I have tried to control diet, my weight has decreased from 105 kg to 93 … (FGD Kupang city).

In terms of diet as a cause, participants explained their experiences that they thought contributed to their dietary habits. As described in Table 3, some participants mentioned that their dietary habits were related to a life event in the past which encouraged them to eat a lot. Participants also said that their dietary habits were part of their culture. Diet in their view, therefore, is not only an individual choice, but is also influenced by their community. This implied further that their efforts to change their diet were also problematic, as it not only depended on their own choices.

**Table 3.** Participants’ narrative concepts of diet causality.

Past life events triggering unhealthy diet [I’ve had sugar disease] since I stopped exercising. Then, I worked as a plastic seller, many customers come in the morning. In the afternoon, almost no one come, so what I’ve done was only eating and eating again … (FGD Solok).
Eating habits as part of culture Yes, we will have a lot of parties in wedding or school season. Sometimes, there are a lot of families in one neighbourhood who do the feast at the same time. Yeah … we will eat a lot, because we can have 10 or perhaps 100 invitations, so, we will eat 10 times or 100 times in a day. That’s why we have high sugar level (FGD Manggarai Timur). We rarely eat vegetables here. What could we do? We are the biggest producers of coconut; everyday coconuts fall down from the tree. If we don’t use it to make *gulai* [yellow curry], we think it’s a waste. We have no choice, it’s like something missing if we don’t make *gulai* (FGD Pariaman 1).
Dietary habits influenced by community changing In the past, we are, people in Manggarai, have a wide range of food diversity. But, all changed … after eating rice become more usual, the other food products are left behind. It was in 1983 (FGD Manggarai Timur).

Moreover, some participants also put the increasing number of people with diabetes into a historical perspective of community eating habits. The participants believed that there was no diabetes in the past as people ate different kinds of food, while today’s generation depends on white rice as a staple food.

Participants also attributed the increase in diabetes to a modernised lifestyle. With the development of transportation systems, people no longer walked. Some participants also mentioned that diabetes was associated with a change in the way they cooked rice. They perceived that the sugar level in rice cooked with a rice cooker is higher than that in rice cooked traditionally. Before rice cookers were popular, people in Indonesia commonly cooked rice slowly in two steps. First, the rice grain was boiled until it was half-cooked. In this step, people often took out the water used for boiling. The half-cooked rice was then steamed for about 30 min. Participants argued that as the sugar is dissolved in the water, taking out the water used for boiling and then steaming the rice reduced the amount of sugar in it. In contrast, they argued that the modern rice cooker eliminated those steps, and consequently, the sugar in rice was believed to be higher.

Long before now, people cooked using the fire woods, then they took the water out, the water was thick, it contains sugar. Therefore, many people suffer from sugar disease because of the modern way of rice cooker (FGD Pariaman).

### Illness experiences

#### Disease progression

Most participants were first diagnosed when their blood glucose reached a very high level that resulted in dramatic consequences such as stroke, or eye surgery. Some participants found out that they had diabetes unexpectedly when they went to healthcare facilities for other health complaints, including blurred vision, gastritis, and asthma.

I just knew that I have sugar when I had stroke. It [sugar level] was 307 [mg/dL]. I’ve had it [stroke] twice … (FGD Pariaman).

Most participants had diabetes for between two and seven years. During this time, they experienced uncontrolled blood glucose, had comorbidities, and developed complications.

He suffered from kidney failure for about 3 to 4 months … Yesterday, the doctor told me that he had to be haemodialysed. I answered, “I don’t know what I have to do”, but the doctor said again, yes mum, he need haemodialysis … (FGD Manggarai).

#### Coping responses

In coping with the disease, at the early stage, some participants denied having diabetes, as they sought second opinions from various health professionals. Others questioned why they got the disease, as they did not experience any symptoms.

I went to a village midwife, and [it was found that] my sugar level was above 500 [mg/dL] (27.8 mmol/L)  …   .But I didn’t trust the result, so I went to another place, [but] it was the same, and I was asked to be hospitalised, but I refused it. Then, I went to the *Puskesmas* and had my sugar level checked (FGD Pariaman 2).

After living with diabetes, most participants shared negative experiences with diabetes-related treatment, such as experiencing uncontrolled blood glucose levels despite the treatment they have done. To cope with this situation, three strategies were identified:

##### Beyond individual’s control

Some participants expressed frustration at not being able to decrease their blood glucose levels. They perceived that their efforts to control diabetes had failed. Therefore, they believed that they could not manage the disease themselves. Some of these participants eventually believed that their disease was caused by a hereditary factor.

Last Saturday I checked my blood sugar, it [blood sugar] used to be normal, however, it increased to 320 … It (blood sugar) can’t be stable, it always fluctuates, we can’t make it balance.. (FGD Pariaman 2)

My sugar level was never getting low … even though I took medicines. I should be not eating at all. I’ve done, I didn’t eat for 3 days, and my sugar level dropped. But, I was really weak. So it can only drop when I don’t eat at all … I’ve substituted rice to corn rice, I do exercise … Perhaps, it’s inherited, have run in our family blood (FGD Manggarai Timur).

They commonly reported barriers in managing the disease, including personal and social-environmental barriers.

Then, we had an event for the elderly, they held activities like a light exercise. Having done that, my sugar level dropped to 121 [mg/dL] (6.7 mmol/L). But I still have to take medicines for the rest of my life, [but] how could I [take the medicines for the rest of my life]? … (FGD Pariaman 1).

We have a lot [community] gathering, and in the gathering all people eat, so we also have to eat. Sometimes, if we do not eat, people will say that we are arrogant, ya that’s people in East Nusa Tenggara (FGD Kupang city).

##### Normalisation of their condition

Some participants coped with the disease through normalising their condition. These people commonly did not consider that high blood glucose levels were serious unless they experienced symptoms. They also had their own interpretation of their ‘normal’ or ‘usual’ blood glucose levels – the levels that did not trigger symptoms or hinder their daily activities.

I usually have [blood sugar level of] 266 [mg/dL]. I just do exercise and drink goat milk, and I feel good [though] it’s 200ish, I could run  … (FGD Pariaman 1)

[F: your blood sugar level is 282 mg/dL] I just ate biscuit, drink coffee, and also ate rice, so, it is good then. I’ve had had it 400 [mg/dL]. For me, I don’t really worry about that, just enjoy  … (FGD Manggarai Timur).

##### Resignation to God

Regardless of specific religion, participants with strong religious beliefs, as shown by frequent references to God during the discussions, commonly applied this coping strategy. These participants also acknowledged that their diabetes was affected by their unhealthy behaviours, and they emphasised the importance of controlling diet, and exercising to manage the disease. In this case, their belief constructed in their narrative did not mean a passive acceptance to fate. Rather, it reflects their submission to God, after all efforts they have made.

Yes, the key point [for managing the disease] is in eating [habits]. [I had experiences] for 4 years, and ya, the key point is in eating [habits]  … and stay calm, [do daily work]: feed cow, chicken, and duck … don’t think too much about that. God has arranged our life (FGD Manggarai Timur).

These participants mentioned their belief that being stressed might worsen their condition, and they saw controlling their mind as an important part of managing the disease. The words ‘*jangan dipikir’*, ‘*jaga pikiran’*, which can be translated as ‘don’t think too much’, or ‘controlling mind’, were often used as expressions for minimising stress. The expression is more relevant to the concept of health as a balance of physical condition and spiritual well-being. With this belief, their emotional burden was minimised.

Yes, so don’t stress, if we are sick and stress, it can deteriorate our condition … (FGD Kupang city).

Calm mind is a medicine, just relax, don’t worry, it’s a medicine. If we think too much, the disease will relapse … (FGD Manggarai Timur).

## Discussion

### Lay perceptions about diabetes

Findings from this study emphasised that people with diabetes constructed illness concepts into their personal narratives based on their experiences. The use of local terms and common lay knowledge about diabetes were also common in other low and middle-income countries (Islam, Biswas, Bhuiyan, Mustafa, & Islam, 2017; Mendenhall et al., 2016; Suparee et al., 2015). The use of local terms, however, may lead to misconceptions of the disease and its symptoms.

Participants in this study connected their beliefs about causality into their own narratives to make sense of why they got the disease. Although it seemed that their logical explanations were in the context of genetic predisposition and lifestyle changes, their personal models differed from the medical model. Lay conceptualisations of diabetes and its causality varies in different cultural contexts (Daniulaityte, 2004; Mercado-Martinez & Ramos-Herrera, 2002; Sowattanangoon, Kotchabhakdi, & Petrie, 2009). For example, people with diabetes in Mexico believed that diabetes was caused by past life events which triggered emotional distress (Daniulaityte, 2004; Mercado-Martinez & Ramos-Herrera, 2002), while people with diabetes in Thailand related diabetes to previous bad action, or bad *karma* (Sowattanangoon et al., 2009).

These personal narratives gave meaning to the individual about their own role in the disease process and its treatment. In our study, participants who perceived genetic inheritance as a cause felt less able to manage the disease. People perceived an inherited disease as a disease that they could not prevent and manage as they got the disease from their ancestor. People who had negative experiences towards diabetes treatments eventually also believed genetic factors as a cause. Therefore, lay concepts of genetic causality have led people to develop a fatalistic attitude, which Davison, Frankel, and Smith (1992) have described as a perception of having not much self-control over their health status. This shows that a personal model of genetic predisposition differed from the medical model, similar to other studies (Pijl et al., 2009; Walter, Emery, Braithwaite, & Marteau, 2004).

In terms of diet as a cause, participants related it to a broader concept of cultural dietary practices. They did not seem to attribute their culture as the reason they had the disease, but they recognised their disease in their cultural context and understood barriers to practising a healthy diet from a broader perspective. Consequently, the wider community needs to be involved in diabetes management. Individuals would be more likely to adopt behaviour if they perceive that it is accepted by their community, and they have enough ability or confidence to do so in a challenging environment (Serrano-Gil & Jacob, 2010).

As part of developing a lay account of diabetes, participants also tried to explain why the number of people with diabetes is increasing, which showed that the participants tried to make sense of their communities’ experience of diabetes. Some participants argued that cooking rice traditionally might reduce the sugar level compared to rice cooked in a rice cooker. No study has been conducted to evaluate differences of the glucose level for rice cooked traditionally and rice cooked in a rice cooker. Some studies have suggested that slowing the rate of starch digestion to glucose could be potentially useful in decreasing the absorbable glucose rate in the small intestine (Svihus & Hervik, 2016). Storing freshly cooked starch-containing food for a prolonged time at cooler temperatures might potentially lower its digestion rate (Svihus & Hervik, 2016; Wang, Li, Copeland, Niu, & Wang, 2015). Therefore, in practice, storing cooked rice in a cooler place for prolonged time could also potential for reducing its digestibility rate. People in Indonesia who cooked rice traditionally often eat the rice cold as the traditional cooker do not have a warmer mode, as in the rice cooker. However, more research needs to be done in this particular area to understand its implications in the Indonesian context.

### Illness experiences

Most participants were diagnosed late, and similar situations have been observed in Iran and India (Abolghasemi & Sedaghat, 2015; Lakshmi, Ganesan, Anjana, Balasubramanyam, & Mohan, 2014). Type 2 diabetes has a long asymptomatic preclinical stage (Porta et al., 2014). Moreover, the general health-seeking behaviour of people in Indonesia, where they do not go to health facilities unless they experience symptoms, further delays diabetes diagnosis. This implies that intervention to increase knowledge and community awareness about diabetes is urgently needed.

After living with diabetes for some period, participants coped with the disease through three strategies: belief that the disease was beyond their control, normalising their condition, and resignation to God. With these strategies, they tried to balance their life and live as normal as possible with their illness. In the shifting perspective model of chronic illness described by Paterson (2001), these strategies were attempts to shift people’s focus of their life from illness to wellness. Studies have also demonstrated various responses of people with diabetes in order to attain balanced life (Campbell et al., 2003).

Strategies of coping influenced people’s behaviours in managing diabetes. In our study, the first and second strategies seemed to have more negative effects to people’s behaviours. People who believed that diabetes was beyond their control, and people who thought that high blood sugar levels were normal unless they experienced symptoms, were more likely to downplay the importance of adherence to diabetes treatment. Fatalistic belief among people with chronic diseases has been suggested as the major cause of non-compliance with lifestyle change and associated with poor diabetes management (Davison et al., 1992; Walker et al., 2012).

The third mechanism of coping is resignation to God. People who used this coping strategy showed a more positive coping response. Spiritual belief does not always mean passive acceptance to fate, but was often a way of finding support in facing stressful life events. Studies involving communities with different spiritual beliefs have shown that individuals with a strong religious belief appeared to have more positive coping responses, as seen by not being frustrated over difficulties as a result of having diabetes (Lundberg & Thrakul, 2013; Rowe & Allen, 2004). Diabetes is a chronic disease that needs persistent effort to manage. Diabetes-related distress might come from its treatment, which includes changing diet pattern, and taking medicines regularly, which is often viewed as burden that interrupts normal life (Wagner & Tennen, 2007). Research has shown that people with diabetes are at risk of depression, and other emotional problems (Polonsky et al., 2005). Furthermore, diabetes-related distress has been found to affect clinical outcomes, and quality of life (Fisher, Glasgow, & Strycker, 2010; Karlsen, Oftedal, & Bru, 2012; Wagner & Tennen, 2007). Therefore, people perceived their religion as a means of coping and a support in the struggle with diabetes (Lundberg & Thrakul, 2013).

### Limitation

Limitations of this study may come from the involvement of two or more languages. Although during the translation process, efforts have been made to increase validity and minimise the risk of losing meaning, there remains a risk that some meaning was lost in some cases.

The study covered a small number of participants in East Nusa Tenggara, and West Sumatera provinces. Therefore, it might not be able to represent populations in other culturally different contexts, or in areas of Indonesia with different characteristics.

### Conclusions

Although people with diabetes attributed their disease to biomedical causes, their personal narratives differed based on their personal situation. Participants also saw the disease in a broader context of cultural identity and environmental changes. These lay concepts influenced their coping strategies and their behaviours in response to diabetes.

### Practice implications

People with diabetes had their own concept about the disease. Therefore, educating people with diabetes should go beyond merely providing information, to internalise the information into people’s narratives and experiences. Further, people’s lay concept of genetic causality may lead the people to develop a fatalistic attitude. Health professionals should be aware of this and be cautious when delivering information about genetic cause of diabetes.

This study also identified cultural and environmental barriers particularly for applying a healthy diet. Therefore, actions for dietary changes should involve the wider community. Involving religious leaders may also potentially support people with diabetes to better manage the disease. Religious practices may further encourage people with diabetes to implement a healthier lifestyle. In one discussion in Kupang city, one participant also mentioned that a pastor also gave advice on healthy lifestyle:

A pastor in a church said to me: we, the people 50 years or older, have to avoid LGG (acronym for *Lemak, Gula, Garam*) fat, sugar and salt. That’s all. Fat for preventing from cholesterol, sugar for diabetes, and salt for hypertension. That’s all.
